# Living on a knife edge-the daily struggle of coping with symptomatic cardiac arrhythmias

**DOI:** 10.1186/s12955-015-0282-9

**Published:** 2015-06-24

**Authors:** Kathleen L Withers, Kathryn A Wood, Grace Carolan-Rees, Hannah Patrick, Mauro Lencioni, Michael Griffith

**Affiliations:** Cedar, Cardiff & Vale University Health Board, Cardiff Medicentre, Heath Park, Cardiff, CF14 4UJ UK; Duke University School of Nursing, 307 Trent Drive, DUMC 3322, Durham, NC 27710 USA; National Institute for Health and Care Excellence, 10 Spring Gardens, London, SW1A 2BU UK; Cardiology Department, University Hospitals Birmingham NHS Foundation Trust, Edgebaston, Birmingham, B15 2GW UK

**Keywords:** Cardiac arrhythmias, Patient reported outcome measures, Qualitative research, Quality of life

## Abstract

**Background:**

In 2010 a retrospective audit was undertaken to assess the viability of using PROMs in patients with symptomatic cardiac arrhythmias having undergone percutaneous arrhythmia ablation. A response rate of 74 % was achieved, with finding suggesting that arrhythmia patients reported a significant impact on their work, social and family life.

**Aims:**

To conduct a qualitative cross sectional survey to understand patients’ perspectives of how cardiac arrhythmias affect their daily lives, as part of a program to develop a Patient Reported Outcome Measure (PROM).

**Method:**

Twenty five patients aged 18 or over, diagnosed with a variety of symptomatic cardiac arrhythmias referred for a cardiac ablation procedure took part in cognitive interviews. These aimed to inform the development of a patient reported outcome measure and to determine factors important to this patient group. Common themes were identified using content analysis.

**Results:**

Participants reported that symptoms of their arrhythmia caused them considerable problems and impacted adversely on their quality of life in many ways. This extended through daily routine, work and social activities and also to friends and family, with fear and anxiety being significant factors for most responders. Patients felt their illness was poorly understood, even by health professionals, and often reported that they felt isolated, lacking support and information.

**Conclusion:**

Symptomatic cardiac arrhythmias are a source of debilitating and life limiting symptoms, having a negative impact on quality of life. Symptoms and related complications are relevant across different arrhythmia substrates and patient groups.

**Trial registration:**

The study is registered on the Clinical Trials website, Identifier NCT01672528

## Introduction

Cardiac arrhythmias are a significant health problem, associated with an increased risk of heart failure, stroke and death [1, 2]. Estimated to affect up to 1 million people a year in the UK they are among the top 10 reasons people go to hospital [3, 4]. This results in a significant burden to the NHS, and studies report that atrial fibrillation (AF) alone is responsible for 6 % of acute medical admissions [5] with the cost of management approximately £459 million in 2000; equating to 1-2 % of NHS expenditure [3, 6].

Arrhythmias cause numerous symptoms including palpitations, breathlessness, syncope and chest-pain, and are linked to reduced quality of life, anxiety and depression [1, 7, 8]. The prevalence of many arrhythmias increases with age, with AF alone occurring in up to 12 % of elderly patients [9, 10]: As the UK population ages, their incidence will increase, with a subsequent impact on the NHS. While treatment primarily remains pharmacological, catheter ablation is now a common treatment option after medicines fail, with guidelines issued by the National Institute of Health and Care Excellence (NICE) on its use [2, 11-14]. Studies report high success rates and low incidence of major complications and mortality [15] and evidence suggests that in many patients ablation may be more beneficial than drug therapy [16, 17].

For most patients, the aim of ablation is to reduce symptoms and improve quality of life. In recent years it has been recognised that patients themselves are best placed to judge clinical benefits of medical procedures, and the use of Patient Reported Outcome Measures (PROMs) has been encouraged [18]. PROMs are questionnaires completed by patients before and after treatment to measure any changes in their health following a medical intervention. While there are various generic PROMs, and numerous questionnaires aimed at patients within specific disease groups, currently no validated disease-specific tools exist for UK patients undergoing catheter ablation of cardiac arrhythmias.

## Background

In November 2010, a retrospective audit was undertaken to assess the viability of using PROMs in patients with symptomatic cardiac arrhythmias having undergone percutaneous arrhythmia ablation. Consecutive patients from three sites were sent: an arrhythmia specific, patient expectation questionnaire; an adaptation of the disease specific “Patient Perception of Arrhythmia” questionnaire; [19] and EQ-5D-5L [20]. In total 791 patients were invited to participate with a response rate of 74 %. Findings indicated many patients quality of life prior to ablation was severely affected by their arrhythmia, with significant impacts on their work, social and family life [21]. Feedback from the audit was used to update and improve the questionnaires.

As part of an iterative process to further develop and validate the questionnaires used in the audit, a multicentre, prospective observational study was designed and approved by Nottingham 1 Research Ethics Proportionate Review Sub-Committee (reference 12/EM/0164). This paper reports on Phase 1 of the study which involved face-to-face cognitive interviews with patients diagnosed with symptomatic cardiac arrhythmias. This aimed to gain insight into factors important to this patient group, identifying core issues of what it is like to live with cardiac arrhythmias. As previous authors have suggested 20 interviews are sufficient to reach data saturation [22, 23], a sample size of 30 patients was defined in the protocol, with fewer to be performed if data saturation was reached earlier.

## Methods

Patients were required to be aged 18+, able to communicate in English or Welsh and previously diagnosed with a symptomatic cardiac arrhythmia, awaiting and/or previously treated with catheter ablation. Purposive sampling was used to include a relevant cross-section of patients (i.e. comprehensive age range, different arrhythmia diagnoses and both sexes).

Patients under the care of clinicians at University Hospital Wales, Cardiff and Queen Elizabeth Hospital, Birmingham were eligible. Consecutive patients who met predefined sampling criteria were invited to participate, and initially approached by their electrophysiology physician who discussed the study and provided written patient information. Individuals interested in participating were subsequently contacted and where appropriate, a mutually convenient appointment for a face-to-face interview made. Twenty-one patients chose to be interviewed in their homes; four opted to be interviewed at a hospital site in a private room. All patients provided written informed consent.

All interviews were conducted by one researcher trained in cognitive interview techniques (KLW). Sessions were tape-recorded with patients’ permission (one patient declined, two were not recorded due to technical issues), with recordings supported by field notes. Interviews used a semi-structured guide based on the draft questionnaire developed by Withers et al. [21] and modified utilising feedback from the 2010 multi-centre audit. Using “think-aloud” techniques participants were instructed to read the questionnaire out loud, and verbalise their thoughts, with the interviewer asking probing questions throughout the process. This enabled identification of potential problems and allowed the researcher to understand how questions were interpreted. Patients were asked to discuss topics lacking clarity within the questionnaire and suggest improvements, they were also asked if there were issues missing from the questionnaire they expected to see, or topics requiring further investigation. At the end of the interview, patients were encouraged to discuss how their arrhythmia affected them, and highlight difficulties in living with a cardiac arrhythmia. Recorded interviews were transcribed verbatim and checked for accuracy.

Interviews followed a grounded theory process of iterative changes, with recurring issues used to inform and guide subsequent interviews. Interviews were carried out until data saturation was reached with no new issues or concerns being identified. Qualitative content analysis with a systematic classification process [24] was used to analyse and code interview data. These were examined to identify recurring themes, with modifications retested in a second phase of interviews. Key quotations were selected to reflect key issues. To ensure rigor, a second researcher experienced in qualitative research was involved in these processes.

### Interview parameters

An initial cohort of 19 patients was interviewed from two ablation centres (n = 13 Birmingham, n = 6 Cardiff), at which point data saturation was reached and revision of the questionnaires undertaken. Following revisions, the questionnaire was re-tested with additional subjects (n = 5 Cardiff, n = 1 Birmingham). Interviews took place between October 2012 and January 2013.

### Patient characteristics

Fifteen (60 %) of the 25 participants were female, and the age range was 43 to 87 years (mean 61 years). The mean average length of time since diagnosis, where known, was 3.24 years (range 11 months-19 years). Fourteen patients (56 %) had received at least one ablation procedure prior to interview, and four of these (16 %) were pending a subsequent procedure. The remaining eleven (44 %) were awaiting their first ablation. Primary arrhythmia diagnoses are detailed in Table 1.

**Table 1:** Primary diagnoses of arrhythmia substrate

	Atrial Fibrillation (AF)*	Supraventricular tachycardia (SVT)**	Atrial Flutter***	Ventricular Ectopics
Female, n (%)	7 (28 %)	5 (20 %)	2 (8 %)	1 (4 %)
Male, n (%)	7 (28 %)	0	3 (12 %)	0

*Two female and one male patients awaiting treatment for paroxysmal AF had already received a previous ablation for paroxysmal AF

**SVT–atrioventricular nodal re-entry tachycardia, accessory pathway, atrial tachycardia

***One female patient awaiting an ablation for persistent atrial flutter had previously received an ablation to treat persistent atrial fibrillation

Eleven participants were retired, and 11 employed; of these, two were on sick leave and three had reduced their working hours due to their arrhythmia. Two participants were not in employment and one was a full time carer.

Recorded sessions lasted between 23 and 93 minutes (mean 50 minutes), with full sessions including study introduction and Q&A lasting between 50 minutes and 4 hours 15 minutes.

## Results

### Everyday impact

All 25 patients (100 %) felt their arrhythmia affected their life every day. This encompassed social and domestic activities as well as voluntary and paid work. For many the random nature of their symptoms prevented them carrying out routine activities such as swimming and dog-walking “just in case” they experienced an episode of palpitations. This had a wide ranging impact for some: “*You stop going shopping, visiting friends or even having a conversation with your family*….”. Others had stopped going out alone as they were too worried about what might happen if they felt unwell. These limitations on patient activities resulted in a feeling of frustration and helplessness, with some stating that they felt alone as a result of their arrhythmia. “*You have a complete feeling of total isolation–you feel like you are the only person in the world who feels like this. You no longer have the chance to have a normal life*”.

For some, arrhythmias were an issue in the workplace with nine patients reporting problems. For these patients this resulted in reducing working hours, rearranging shifts or even changing jobs to try and reduce physical or mental stress, which participants often felt was a causative factor for triggering further episodes of palpitations. “*I gave up my job as it was stressful and I think this made my arrhythmia worse*”. Several patients commented that they “covered up”, by attempting to hide symptoms from colleagues, as they were concerned that it might affect their employment. In two cases patients had retired from work earlier than planned as they felt unable to cope with their unpredictable and wide-ranging symptoms. “*I retired although I didn’t want to, but going to work became too hard*”.

### Impact on family life

Symptoms reported by the study group were more far-reaching than anticipated from the literature, with responses illustrating a significant perceived impact on family members and this placed a subsequent pressure on some relationships. Several patients had stopped caring for grandchildren with a resultant financial implication for family members. For others, reliance on friends and family to help with tasks such as shopping left them feeling guilty and led to a loss of independence. This resulted in a degree of withdrawal from family contact for those affected. “*I won’t have my grandchildren on my own anymore in case I have an episode, and my husband will go out without me because I don’t feel up to doing anything*”. Some patients reported withholding information about the severity and regularity of symptoms from family members as “*I don’t want to worry them even more*”. Inevitably this exacerbated their feeling of loneliness and stress from strained relationships.

### Fear and anxiety

Many of the patients interviewed regarded the most important problem as being the almost constant fear and anxiety that they reported experiencing, with 80 % of responders describing this as an issue. Whilst some had been told the risks associated with their condition were small, involvement of their heart was still a significant concern. The physical symptoms frightened many, leading to an emotional burden they found difficult to deal with. “*The fear NEVER goes away. Worry is not a strong enough word…..I feel panic stricken ALL the time*”. Some worried their heart-rhythm problems would damage their overall health and were afraid they would have a heart-attack or stroke every time their palpitation started. “*The fear is worse than anything because they can come at any time. I am constantly living in terror and frantic with fear*” and “*It’s like living on a knife edge*”. Some patients felt overwhelmed by their illness: “*Every day I wake up worrying. It stops me leading a normal life–I’m afraid to be on my own in case I get an episode*”.

### Getting the right support and information

The level of information and support received varied considerably, and some participants felt their primary care physician/GP was unable to deal with their arrhythmia and did not take their symptoms seriously. One patient reported that her GP had told her “*You just need a good kick up the backside*”. Several others felt that they were not believed pre-diagnosis due to the lack of obvious symptoms and difficulties in recording the arrhythmia to get a definitive diagnosis. Lack of forthcoming practical advice in the form of background information, and how to try and record the episodes or how to evidence and manage them left some patients we spoke to unsure where to go for support and advice.

Following diagnosis some participants felt they received insufficient information about arrhythmias, adding to their worry. Several suggested the availability of more information would have been reassuring. “*The worst thing was the lack of information....nothing was forthcoming. During that time anxiety levels are high because you need more information*”.

Several participants found online support groups useful, leading to patients who had used them suggesting these could be more widely utilised. Written information such as patient booklets advising on symptoms, treatment and limitations was also a resource some responders felt would have been helpful. “*The doctor told me I had AF–well that meant nothing to me. You then get discharged with nothing at all…….if they explained you could pick up a booklet from your GP....,but instead you are sent back home without knowing what to do*”. In some cases lack of information extended to the procedure and outcomes, and this was occasionally a significant issue: “*I wasn’t told that it might not work, and definitely no mention that my symptoms could get worse. I felt so low after the procedure because I expected to get better but felt worse, to be honest it made me feel like ending it all*”.

Other participants felt they had received useful information, readily supplied at the ablation clinic. This made them more confident about what to expect with relation to their condition and care and this reduced anxiety levels. “*I had good information from my consultant on what to expect after my procedure which was very helpful*”. Most patients involved were unaware the procedure was not guaranteed to work while others conceded they were unable to remember everything they were told in clinic appointments. This led to some participants suggesting that written information would be helpful, as would details for support groups. Where available, arrhythmia nurse specialists were reported to be a helpful contact point for information.

### Successful procedure

A number of post ablation participants suggested they had only realised the full impact of their arrhythmia following successful treatment having previously put some symptoms down to aging. These patients were surprised by their improvements following ablation. “*It was a shock to me…….I got used to being tired, and used to being breathless. It was gone after the operation–it was a shock when it stopped*” and “*I didn’t realise that’s what caused it …… it’s not my age*!” Those patients interviewed where ablation had abolished or reduced symptoms were very happy with the outcome and improvements it had made to their lives: “*I know that heart ablation has transformed my life for the better and I am very grateful*”. In general, even those awaiting a further ablation were positive about their experience and were still optimistic about the procedure.

## Discussion

### Qualitative themes

Our findings highlight some of the hurdles that patients with cardiac arrhythmias can face in managing symptoms and living with uncertainty. It is important for health professionals to appreciate the challenges these patients face to assist them in managing their condition. Themes identified in our data demonstrate that at least for some patients, arrhythmias have a significant negative effect on their lives; leads to a perceived reduction in overall quality of life; impacts all aspects of their life: work, social, and daily activities; results in almost constant fear and anxiety; and requires enormous time to search for support and information. Walfridsson and colleagues [25] also noted SVT patients’ decreased engagement with social activities. Research has also found patients with SVT [26] and AF [27] report feeling uninformed, unsupported, and distressed. Our study found that while most participants felt supported by those close to them, some experienced a lack of support from the medical community, feeling they were often thought to be “making a fuss”. Woods and colleagues [26] also found that health-care providers were noted to minimize patient complaints of symptoms and offered little support, with patients feeling disbelieved when physicians ignored their descriptions of symptoms when no tachycardia was found on intermittent monitoring. These researchers also reported that the patient-provider relationship suffered when the patient perceived disbelief on the providers’ behalf. When patients were finally correctly diagnosed it was seen as a relief that they were believed and could now progress to seeking corrective treatment.

While others have also found that managing life with an arrhythmia is formidable, our findings add new insight into how alone some patients feel in their struggle for information and how providers may be perceived as offering little support with symptom management, their need for more detailed information about their condition, and desired help with decision making regarding treatment options. Increased emotional distress and feelings of uncertainty related to arrhythmia have been reported by others [26, 28]; however, our study also found that some patient felt that their arrhythmia cause a negative impact on family life and led to feelings of isolation, which has been poorly described in previous literature. Our study found 40 % of participants reported a perceived negative impact on family and close friends due to their condition, often leading to feelings of guilt and further isolation for the patient as they felt compelled to withdraw from some family and social occasions. For those who no longer felt able to care for grandchildren “just in case” they became unwell, this guilt was exacerbated. This was also true for those patients forced to change jobs or retire early due to the impact of their arrhythmia, causing a detrimental economic impact for the family.

### Strengths and limitations

While the interviews involved a range of patients, the sample size was relatively small and it is difficult to be sure the cohort is representative of the patient population. Different ethnic groups and socio-economic factors may result in patients with differing issues to those covered in this study. Some patients invited to interview declined, and potentially this sub-group share different characteristics and inherent problems to those who participated. We strengthened the study by inviting consecutive patients, and by having a single researcher conducting all interviews for consistency. Additional steps taken to ensure quality and trustworthiness of data analysis included the following: all transcribed interviews were checked by the interviewer for consistency and all audio-taped interviews were checked line by line for accuracy. An audit trail of definitions of data categories and coding decisions was maintained, and a second researcher involved in data analysis. Transferability was achieved by sampling as diverse a group of participants as possible.

## Conclusion

This study of participants’ first-hand experience of living with cardiac arrhythmias provides useful insight into factors that are most important in the patient experience. Although several types of arrhythmia were involved in this study, common themes were apparent, particularly with regard to the fear involved with living with their condition and the concern that long-term harm would occur. This study illustrates the unpredictability of symptoms is a significant burden not only to the patient, but also to those closest to them. The unpredictable nature of symptoms led to major lifestyle changes for patients within the study, in the belief that aspects of their normal routine might trigger episodes of palpitations, or even be harmful to their heart. This had a detrimental outcome far beyond the scope of the symptoms of the condition, leading to reduced physical activity and social interaction, furthering the sense of isolation. Involvement of the heart, no matter how insignificant from a clinical viewpoint is a major concern for patients. During early consultations, it is essential that clinicians spend time explaining the condition and its potential impact to patients to ensure they are aware of the limitations imposed by arrhythmias. The support of specialist arrhythmia nurses is appreciated by patients, and use of written patient education material and comprehensive advice and support can inform and reassure patients and help to provide realistic expectations and limitations. Patient experience data is a valuable resource and can be used to improve the quality of health care by identifying strengths and weaknesses of current provision.

## References

[1] Camm JA, Kirchhof P, Lip GYH (2010). Guidelines for the management of atrial fibrillation. The task force for the management of atrial fibrillation of the European society of cardiology (ESC). Eur Heart J.

[2] National Institute for Health and Clinical Excellence (2009). Percutaneous (non-thorascopic) epicardial catheter radiofrequency ablation for atrial fibrillation. NICE Interventional Procedure Guidance 294.

[3] DH Coronary Heart Disease Team (2005). National Service Framework for Coronary Heart Disease. Chapter Eight: Arrhythmias and Sudden Cardiac Death.

[4] NHS Choices (2013). Heart rhythm problems. NHS Choices.

[5] Lip GYH, Tean KN, Dunn FG (1994). Treatment of atrial fibrillation in a district general hospital. Br Heart J.

[6] Stewart S, Murphy N, Walker A (2004). Cost of an emerging epidemic: an economic analysis of atrial fibrillation in the UK. Heart.

[7] Thrall G, Lane D, Carroll D, Lip GYH (2006). Quality of life in patients with atrial fibrillation: A systematic review. Am J Med.

[8] Thrall G, Lip GYH, Carroll D, Lane D (2007). Depression, anxiety, and quality of life in patients with atrial fibrillation. Chest.

[9] Lok NS, Lau CP (1996). Prevalence of palpitations, cardiac arrhythmias and their associated risk factors in ambulant elderly. Int J Cardiol.

[10] Wolf PA, Abbott RD, Kannel WB (1991). Atrila fibrillation as an independent risk factor for stroke: the Framington study. Stroke.

[11] National Institute for Health and Clinical Excellence (2006). Percutaneous radiofrequency ablation for atrial fibrillation. NICE interventional procedure guidance 168.

[12] National Institute for Health and Clinical Excellence (2006). Atrial fibrillation. NICE clinical guideline 36.

[13] National Institute for Health and Clinical Excellence (2011). Percutaneous endoscopic catheter laser balloon pulmonary vein isolation for atrial fibrillation. NICE Interventional Procedure Guidance 399.

[14] National Institute for Health and Clinical Excellence (2012). Percutaneous balloon cryoablation for pulmonary vein isolation in atrial fibrillation. NICE interventional procedure guidance 427.

[15] De Loma-Osorio AF, Diaz-Infante E, Gallego AM (2013). Spanish catheter ablation registry. 12th official report of the spanish society of cardiology working group on electrophysiology and arrhythmias (2012). Rev Esp Cardiol.

[16] Jais P, Cauchemez B, Macle L (2008). Catheter ablation versus antiarrhythmic drugs for atrial fibrillation: The A4 study. Circulation.

[17] Pappone C, Vicedomini G, Augello G (2011). Radiofrequency catheter ablation and ANtiarrhythmic drug therapy: a prospective, randomised 4-year follow up trial: the APAF study. Circulation Arrhythmia Electrophysiol.

[18] Department of Health (2010). Equity and excellence: liberating the NHS.

[19] Wood KA, Stewart AL, Drew BJ (2009). Development and initial psychometric evaluation of the patient perspective of arrythmia questionnaire. Res Nurs Health.

[20] The EuroQol Group (1990). EuroQol-a new facility for the measurement of health-related quality of life. Health Pol.

[21] Withers K, White J, Carolan-Rees G (2014). Patient reported outcome measures for cardiac ablation procedures-a multicentre pilot to develop a New questionnaire. Europace.

[22] Guest G, Bunce A, Johnson L (2006). How many interviews are enough? an experiment with data saturation and variability. Field Methods.

[23] Kerr C, Nixon A, Wild D (2010). Assessing and demonstrating data saturation in qualitative inquiry supporting patient reported outcomes research. Expet Rev Pharmacoeconomics Outcome Res.

[24] Hsieh HF, Shannon SE (2005). Three approaches to qualitative content analysis. Qual Health Res.

[25] Walfridsson U, Walfridsson H (2005). The impact of supraventricular tachycardias on driving ability in patients referred for radiofrequency ablation. PACE.

[26] Wood KA, Wiener CL, Kayser-Jones J (2007). Supraventricular tachycardia and the struggle to be believed. Eur J of Cardiovasc Nurs.

[27] McCabe PJ, Schumacher K, Barnason SA (2011). Living with atrial fibrillation: a qualitative study. J Cardiovasc Nurs.

[28] McCabe PJ (2011). What patients want and need to know about atrial fibrillation. J Multidiscip Healthc.

